# Beyond neurons: Impact of cannabidiol on glial cells in ischemic stroke

**DOI:** 10.4103/NRR.NRR-D-25-01029

**Published:** 2026-01-27

**Authors:** Victória Linden de Rezende, Khiany Mathias, Cinara Ludvig Gonçalves, Rafael Mariano de Bitencourt, Tatiana Barichello, Fabricia Petronilho

**Affiliations:** 1 Laboratory of Experimental Neurology, Graduate Program in Health Sciences, University of Southern Santa Catarina (UNESC), Criciúma, SC, Brazil; 2 Behavioral Neuroscience Laboratory, Postgraduate Program in Health Sciences, University of Southern Santa Catarina (UNISUL), Tubarão, SC, Brazil; 3 Faillace Department of Psychiatry and Behavioral Sciences, Translational Psychiatry Program, McGovern Medical School, The University of Texas Health Science Center at Houston (UTHealth), Houston, TX, USA

**Keywords:** blood–brain barrier, cannabidiol, endocannabinoid system, glial cells, ischemic stroke, neuroinflammation, neuroprotection, oxidative stress

## Abstract

Ischemic stroke triggers a complex cascade of events involving inflammation, oxidative stress, and glial cell dysfunction, all of which contribute to neuronal damage and impaired recovery. Glial cells (e.g., astrocytes, microglia, and oligodendrocytes) play key roles in neuroinflammatory responses, making them attractive targets for therapeutic modulation. Cannabidiol, a non-psychoactive phytocannabinoid from *Cannabis sativa*, exhibits anti-inflammatory, antioxidant, and neuroprotective properties. Preclinical evidence indicates that cannabidiol attenuates glial reactivity, reduces pro-inflammatory signaling, mitigates oxidative stress, and preserves blood–brain and intestinal barrier integrity in stroke models. Moreover, cannabidiol modulates key molecular pathways (e.g., nuclear factor-κB, tumor necrosis factor, and calcium-related signaling), contributing to reduced infarct volume and improved neurological function. Despite these promising effects, clinical translation is hindered by a lack of standardized formulations, dosing regimens, and human trials. This review highlights the impact of cannabidiol on glial cell activity in ischemic stroke, proposing it as a multi-target agent with therapeutic potential in post-stroke recovery and neuroprotection.

## Introduction

The endocannabinoid system (ECS) is a complex biological signaling network present in various tissues, with a prominent role in the central nervous system (CNS). It consists of endocannabinoids, their primary cannabinoid receptor type 1 and 2 (CB1R and CB2R), and the enzymes responsible for their synthesis and degradation. The ECS regulates several physiological processes, including neurotransmission modulation, immune response, inflammation, and cellular homeostasis, playing a crucial role in maintaining brain health (Zou and Kumar, 2018; Chayasirisobhon, 2021).

Among the phytocannabinoids derived from the *Cannabis sativa* plant, cannabidiol (CBD) has received significant attention due to its versatile pharmacological properties, including anti-inflammatory, antioxidant, neuroprotective, and immunomodulatory actions (Atalay et al., 2019; Martinez Naya et al., 2023, 2024). Unlike Δ9-tetrahydrocannabinol (THC), the plant’s primary psychoactive compound, CBD does not exhibit significant psychoactive effects, making it a promising candidate for therapeutic applications (Martinez Naya et al., 2024). CBD exerts its effects by modulating the ECS and interacts with various ion channels and receptors involved in neurophysiological and immunological processes, such as transient receptor potential vanilloid 1 (TRPV1), peroxisome proliferator-activated receptor gamma (PPARγ), and 5-hydroxytryptamine receptor 1A (5-HT1A) (Hayakawa et al., 2010; De Petrocellis et al., 2011; Martinez Naya et al., 2023).

Glial cells, including astrocytes, microglia, and oligodendrocytes, are essential for brain homeostasis, participating in the maintenance of the blood-brain barrier (BBB), providing metabolic support to neurons, regulating neuroinflammation, and orchestrating immune responses in the CNS (Bokobza et al., 2019; Paolicelli et al., 2022). During pathological events such as ischemic stroke (IS), these cells undergo morphological changes, contributing both to repair mechanisms and to inflammatory responses that may exacerbate neuronal damage (Sofroniew and Vinters, 2010). Therefore, modulation of glial activity represents a key strategy for controlling neuroinflammation and promoting neuroprotection.

In this context, CBD has been investigated as a potential agent capable of modulating glial cell function, exhibiting anti-inflammatory and antioxidant effects that may attenuate the exacerbated glial response following stroke (Atalay et al., 2019; Somensi et al., 2019). Experimental models of cerebral ischemia indicate that CBD reduces microglial and astrocytic activation, decreases the expression of pro-inflammatory mediators such as nuclear factor kappa-light-chain-enhancer of activated B cells (NF-κB) and tumor necrosis factor (TNF), and protects against oxidative stress, thereby supporting functional recovery after injury (Castillo et al., 2010; Bigdeli and Khaksar, 2017; Khaksar and Bigdeli, 2017).

Current therapies for IS focus on restoring cerebral blood flow through intravenous thrombolysis or mechanical thrombectomy, but their use is limited by a narrow therapeutic window (Campbell et al., 2019; Berge et al., 2021; Mosconi and Paciaroni, 2022). Several molecules, including minocycline (Lampl et al., 2007; Srivastava et al., 2012; Pawletko et al., 2023), PPAR-γ agonists (Han et al., 2015; Li et al., 2025), and nicotinamide adenine dinucleotide phosphate oxidase inhibitors (Wang et al., 2006), have been investigated to reduce inflammation, oxidative stress, and neuronal damage after stroke. CBD has emerged as a potential therapeutic agent due to its ability to modulate glial activation, reduce neuroinflammation, and promote functional recovery in preclinical models (Ceprián et al., 2017; Mori et al., 2017; Yokubaitis et al., 2021; Meyer et al., 2022; Raïch et al., 2024).

Therefore, this review aims to explore the role of CBD in modulating glial cells in the context of stroke, analyzing the underlying molecular mechanisms and discussing the therapeutic implications of this interaction for neuroprotection and post-stroke recovery.

## Search Strategy

The literature search was conducted between May and October 2025 in PubMed (via NCBI), Scopus (via Elsevier), and Web of Science Core Collection (via Clarivate). Peer-reviewed original and review articles published in English were considered. The search strategy included the following keywords in various combinations: cannabidiol, CBD, glial cells, astrocytes, microglia, oligodendrocytes, ischemic stroke, neuroinflammation, and neuroprotection.

## Cannabidiol: General Aspects

*Cannabis sativa* is an indigenous plant that synthesizes a variety of chemical compounds with several medicinal properties, including analgesic, anti-inflammatory, immunosuppressive, anticonvulsant, and antiemetic effects (Friedman and Devinsky, 2015; Chayasirisobhon, 2021). Despite these benefits, harmful effects have also been observed, such as alterations in cognitive function and changes in signal transduction within the sympathetic and parasympathetic nervous systems (Chayasirisobhon, 2021). Among the compounds derived from cannabis, the most abundant are THC, CBD, terpenes, and flavonoids. It is important to note that THC is the main psychoactive compound in cannabis, while CBD is the primary non-psychoactive component (Martinez Naya et al., 2024). In this context, CBD has gained attention due to its diverse therapeutic potential, showing promising anti-inflammatory and analgesic effects (Martinez Naya et al., 2024).

CBD was first isolated in the late 1930s and early 1940s; however, its structure was only elucidated in 1963 (Mechoulam et al., 2002). CBD is classified as a phytocannabinoid and is composed of a terpenophenolic structure, containing twenty-one carbon atoms arranged in a cyclohexene ring, a phenolic ring, and a pentyl side chain (Atalay et al., 2019). It is also important to note that CBD shares the exact same molecular formula as THC, which is C_10_H_30_O_2_. However, there is a slight structural difference between the two compounds: THC contains a cyclic ring, whereas CBD has a hydroxyl group (Jones et al., 1977). Due to its small size and lipophilic nature, CBD tends to accumulate in fat-rich regions, remaining temporarily stored in adipose tissue. It then penetrates highly vascularized areas, rapidly reaching organs such as the brain, liver, heart, and lungs, resulting in a rapid decline in its blood levels (Lucas et al., 2018). It is important to emphasize that CBD is an exogenous phytocannabinoid that modulates the ECS but is not an intrinsic component of it (Boggs et al., 2018).

The ECS is a complex signaling network composed of endogenous cannabinoids (endocannabinoids), their receptors, primarily CB1R and CB2R, and the enzymes responsible for their synthesis and degradation (Martinez Naya et al., 2023). This system plays a crucial role in maintaining physiological homeostasis by modulating processes such as pain, mood, appetite, immune function, and neuroinflammation (Zou and Kumar, 2018). CBD exerts its therapeutic effects via the ECS by interacting with G protein-coupled receptors, acting as a negative allosteric modulator at CB1R, while its effects on CB2R are primarily indirect, mediated by increased endocannabinoid availability and related modulatory pathways (Laprairie et al., 2015; Zou and Kumar 2018). Unlike orthosteric ligands that bind directly to the active site of a receptor, CBD binds to an allosteric site on CB1R, modulating its response to endogenous agonists without directly activating the receptor (Laprairie et al., 2015; Pandey et al., 2025). In addition to modulating these classical receptors, CBD interacts with various ion channels and G protein-coupled receptors, such as TRPV1, TRPV2, TRPV3, TRPV4, TRPA1, and transient receptor potential melastatin 8 (TRPM8) channels, as well as the GPR55, GPR18, and PPARγ receptors (De Petrocellis et al., 2011; Martinez Naya et al., 2023).

The CBD has demonstrated significant anti-inflammatory, antioxidant, and neuroprotective properties, many of which are associated with its interaction with components of the ECS and related receptors (Martinez Naya et al., 2023). Interestingly, several studies have demonstrated the effects of CBD in various conditions affecting the central nervous system, including its ability to modulate neuroinflammatory processes, reduce oxidative stress, and promote neuroprotection in conditions such as epilepsy, autism, IS, multiple sclerosis, Parkinson’s disease, and Alzheimer’s disease (Costa et al., 2025; de Pieri Pickler et al., 2025; de Souza Stork et al., 2025). Numerous studies are being conducted in the preclinical field to better understand the therapeutic mechanisms associated with CBD. A recent study by Costa et al. (2025) showed that rats subjected to the valproic acid-induced autism model exhibited behavioral and biochemical deficits that were reversed by treatment with a combination of CBD and risperidone, indicating potential therapeutic effects. Furthermore, extracts containing not only CBD but also minor cannabinoids (e.g., Cannabigerol, Cannabichromene, THC in trace amounts < 0.3%), terpenes, flavonoids, and other natural phytochemicals were shown to improve neurological deficits in animals subjected to the middle cerebral artery occlusion (MCAO) model. Additionally, the authors observed a decrease in blood cells related to the immune response, reduced atrophy of lymphoid organs, as well as a decrease in intestinal barrier permeability (de Souza Stork et al., 2025). In the context of clinical trials, positive effects of CBD treatment have been observed in improving the quality of life of individuals with Parkinson’s disease (Chagas et al., 2014), as well as in reducing the frequency of epileptic seizures (Devinsky et al., 2016).

## Glial Cells and Cannabidiol: Roles in Physiology and Disease

### Cannabidiol and astrocyte function

The name “astrocytes” comes from the Greek astro, meaning “star,” in reference to their star-shaped appearance. In the late 19^th^ and early 20^th^ centuries, Ramón y Cajal and Camillo Golgi had already observed this characteristic, although the morphology of astrocytes is quite distinct (Kettenmann and Verkhratsky, 2011; Gradisnik and Velnar, 2023). It is estimated that astrocytes make up 25% to 50% of the total volume of brain tissue, highlighting their role as extremely important components in the architecture of the CNS (Bedner et al., 2020; Gradisnik and Velnar, 2023).

According to advances in cellular biology and physiology studies, the role of astrocytes in brain function has become clearer, and today it is known that these cells perform important functions in both healthy and pathological conditions (Carmen et al., 2007). Astrocytes contribute to axonal guidance, stimulation of neuronal growth, synapse formation, transfer of metabolites between blood vessels and neurons, myelination, and maintenance of the BBB, among other functions (Zupan et al., 2000; Bokobza et al., 2019). Additionally, these cells also express damage-associated molecular patterns (DAMPs) and pathogen-associated molecular patterns (PAMPs) (Liddelow et al., 2017), and under normal conditions, they release a growth-modulating factor (transforming growth factor-beta) that promotes an anti-inflammatory environment. However, in response to infection or injury, astrocytes release pro-inflammatory cytokines following microglial activation, thus participating in neuroinflammation (Bokobza et al., 2019).

During pathological events, astrocytes undergo a process known as astrogliosis, in which these cells proliferate, undergo morphological changes, and exhibit altered gene expression (David et al., 2009; Sofroniew and Vinters, 2010; Matusova et al., 2023). These activated astrocytes form a barrier at the edges of the lesion areas, initially protective, but which can evolve into a glial scar that hinders neuronal regeneration (Sofroniew, 2009). Furthermore, their function in maintaining the BBB can be compromised, allowing infiltration of immune cells from the periphery, thereby increasing inflammatory damage (Sofroniew and Vinters, 2010). It is also important to highlight the role of these reactive cells in activating inflammatory pathways, such as NF-κB, which promotes changes that increase the expression of pro-inflammatory genes and repress homeostatic genes, contributing to a chronic and harmful response (Brambilla et al., 2005; Sofroniew, 2009). Functionally, astrocytes lose the ability to uptake glutamate and transport potassium, resulting in ionic imbalances and excitotoxicity, as well as reducing lactate production, which is essential for the metabolic support of neurons (David et al., 2009; Sofroniew and Vinters, 2010; Robel et al., 2015). Thus, it is understood that although astrocytes play fundamental roles in support and protection in healthy CNS, their exaggerated and chronic response can worsen neurodegeneration and hinder post-injury recovery.

In this scenario, substances with the potential to modulate astrocyte reactivity have gained prominence as possible therapeutic strategies. Among them, CBD, one of the main non-psychoactive compounds of the *Cannabis sativa* plant, has been investigated for its anti-inflammatory and neuroprotective effects (Gómez del Pulgar et al., 2002; Tarassishin et al., 2014; Atalay et al., 2019; di Giacomo et al., 2020). Several studies have demonstrated the beneficial effects of CBD on astrocytes under conditions of oxidative stress and inflammation. CBD has been shown to effectively reduce the production of reactive oxygen species (ROS) in astrocytes exposed to hydrogen peroxide, possibly due to its ability to chelate transition metal ions, such as iron, which are involved in the Fenton reaction, a process responsible for generating highly reactive hydroxyl radicals (Atalay et al., 2019; di Giacomo et al., 2020). This antioxidant effect was accompanied by a reduction in apoptosis, associated with the modulation of pro-apoptotic proteins such as Bax and cleaved caspase (Gómez del Pulgar et al., 2002; di Giacomo et al., 2020). In inflammatory models, CBD inhibited the pro-inflammatory responses of astrocytes and microglia stimulated with lipopolysaccharide (LPS), significantly reducing the phosphorylation of signal transducer and activator of transcription 3 and NF-κB, key pathways in the induction of inflammatory cytokines such as TNF, interleukin-1α (IL-1α), interleukin-1β (IL-1β), and interleukin-6 (IL-6) (Tarassishin et al., 2014; Somensi et al., 2019; Wu et al., 2021).

Furthermore, although CBD reduced IL-6 release, it had no significant effect on TNF release. Other findings show that CBD can also inhibit the formation of neurotoxic reactive astrocytes, reduce gliosis, and prevent neuronal loss in murine models of kainic acid-induced epilepsy, both *in vitro* and *in vivo* (Ye et al., 2025). In these models, CBD treatment regulated the expression of the adenosine A2A receptor, reduced the release of inflammatory cytokines, and reversed lipid accumulation in astrocytes. In a more recent study, CBD was found to improve the energy metabolism of astrocytes stimulated by LPS, promoting increased glycolysis, reduced mitochondrial proton leakage, and greater coupling efficiency effects mediated by CB1R, with a consequent reduction in ROS and cytokines such as IL-6 and TNF (Ibork et al., 2023).

### Cannabidiol as a modulator of microglial activity

Microglia comprise the resident immune cells of the brain and spinal cord (Kierdorf et al., 2013). During synaptogenesis, they are responsible for inducing apoptosis in excess neurons, as well as contributing to the regulation of synapse formation (Bessis et al., 2007). Additionally, they are responsible for myelination, neurogenesis, neural function, vasculogenesis, maintenance of BBB permeability, as well as inflammation (Paolicelli et al., 2022). This is the first type of glial cell to respond to pathological changes in the brain, releasing cytokines and prostaglandins that will recruit astrocytes to the site of injury, contributing to the exacerbation of the inflammatory process (Kreutzberg, 1996). Microglia possess various types of cytokine and chemokine receptors, in addition to DAMPs and PAMPs, which help this cell group detect environmental changes (Biber et al., 2014). Interestingly, studies have shown that microglia not only respond to local signals within the brain but also receive continuous input from the periphery, including signals originating from the gastrointestinal tract (Abdel-Haq et al., 2019; Erny et al., 2021). Moreover, there is the phenomenon known as “sickness behavior,” a microglial response triggered by the detection of PAMPs produced by peripheral immune cells and resident tissue macrophages (Dantzer, 2009).

In an inflammatory context, microglia sense environmental changes through their Toll-like receptors, which are transmembrane receptors that detect PAMPs and DAMPs (Matzinger and Kamala, 2011; Muzio et al., 2021). Activation of Toll-like receptor-associated signaling pathways leads to the production of pro-inflammatory cytokines or the induction of type I interferons, resulting in the release of interferon-β and chemokines (Kawai and Akira, 2010). Thus, microglia are rapidly stimulated following a series of pathological events, including altered neuronal function, infection, injury, ischemia, and inflammation. In response to these factors, microglia undergo a morphological transition to an amoeboid state, facilitating their migration to the site of insult (Thameem Dheen et al., 2007; Smith et al., 2012). The cytotoxic mediators released by reactive microglia include reactive oxygen and nitrogen species (superoxide anion, nitric oxide), excitotoxic glutamate, and histamine, all of which may contribute to a neuroinflammatory state in the CNS (Chang et al., 2024; Zhou et al., 2024; Dash et al., 2025). Additionally, it is important to note that microglia also act as mediators of neuroprotection, as they are a source of neurotrophic factors such as nerve growth factor, brain-derived neurotrophic factor, and neurotrophin-4/5. Thus, it is understood that these glial cells play a neuroprotective role in the short term, while their chronic stimulation is implicated as a potential mechanism in neurodegenerative disorders (Flury et al., 2025; Valiukas et al., 2025).

Recent studies have highlighted the therapeutic potential of CBD in neurological and neuroinflammatory contexts. In an *in vitro* model of kainate-induced seizures in hippocampal cells, CBD demonstrated dose-dependent neuroprotective effects by reducing cell death in the CA3 sub-region and inhibiting microglial reactivity through the activation of TRPV1, TRPV2, 5-HT1A, and PPARγ receptors (Landucci et al., 2022). Similarly, in HC69.5 (immunodeficiency virus/GFP^+^) human microglial cells infected with the human immunodeficiency virus, CBD significantly reduced the expression of inflammatory cytokines such as IL-6, IL-8, monocyte chemoattractant protein-1, C–X–C motif chemokine ligand 1, C–X–C motif chemokine ligand 10, and IL-1β, inhibited the inflammasome pathway (NLRP3/caspase-1), and decreased viral expression when compared to Δ(9)-THC treatment (Yndart Arias et al., 2023). Additionally, in the context of Alzheimer’s disease, CBD enhanced microglial phagocytosis of amyloid-β peptide through TRPV2 receptor activation, improving autophagy, mitochondrial function, and adenosine triphosphate production, while simultaneously attenuating neuroinflammation in both murine and human models of Alzheimer’s disease (Yang et al., 2022). These findings support the role of CBD as a multi-target agent capable of modulating neuroinflammation and protecting against neuronal damage caused by pathological conditions of the central nervous system.

### Impact of cannabidiol on oligodendroglial cells

Oligodendrocytes are the glial cells responsible for the myelination process during the development of the CNS (Franklin and ffrench-Constant, 2017), and their precursors are the neural progenitor cells, present in the neural tube during embryonic development (Davis and Temple, 1994). The myelin sheath produced by oligodendrocytes acts as an electrical insulator and facilitates the transmission of nerve impulses through saltatory conduction, a process in which the action potential “jumps” from one node of Ranvier to the next (Kuhn et al., 2019). This occurs because membrane depolarization happens only at the nodes, due to the low capacitance of the myelin sheath, allowing faster and more efficient propagation of the electrical signal along the axon (Nave, 2010; Kuhn et al., 2019).

Interestingly, other functions of oligodendrocytes in the CNS can also be highlighted, including the synthesis and release of lactate, which can be transferred to axons to generate energy in the form of adenosine triphosphate (Bonora et al., 2014; Kuhn et al., 2019). Additionally, it is worth noting that oligodendrocyte precursor cells (OPCs) possess immunomodulatory capacity, as they express cytokine receptors and act as cross-presenting antigen-presenting cells to cytotoxic CD8^+^ T cells (Falcão et al., 2018). Under pathological conditions, such as oxidative stress, oligodendrocytes and OPCs are highly vulnerable due to their reduced antioxidant capacity and high iron content (Butts et al., 2008; Kim et al., 2020). Additionally, they are susceptible to cytotoxic byproducts and to excitotoxicity caused by elevated concentrations of glutamate and adenosine triphosphate. Thus, these cells are highly sensitive to various forms of injury, including trauma, ischemia, and autoimmune diseases, which can lead to their death and, consequently, impair the myelination process (Matute et al., 2007; McTigue and Tripathi, 2008).

Growing evidence indicates that the ECS, including CBD, exerts protective and modulatory effects on oligodendroglial cells, especially under conditions of stress and inflammation (Mecha et al., 2012; Ilyasov et al., 2018; Manterola et al., 2022). CBD reduces the production of ROS, inhibits LPS/interferonγ-induced apoptosis, and attenuates cell death associated with endoplasmic reticulum stress, while promoting the expression of anti-apoptotic genes such as Bcl-2, independently of CB1, CB2, TRPV1, and PPARγ receptors (Mecha et al., 2012). Furthermore, cannabinoids promote the survival, proliferation, and differentiation of OPCs, supporting processes of myelination and remyelination (Ilyasov et al., 2018). Complementing these functional findings, morphological studies have demonstrated the presence of CB1 receptors in adult hippocampal OPCs, albeit at low density, providing anatomical support for the hypothesis that cannabinoids act directly on these cells and influence myelin dynamics in the adult brain (Manterola et al., 2022).

## Implications of Glial Effects of Cannabidiol in Ischemic Stroke Context

### Basic concepts of stroke

IS is defined as an acute and focal neurological deficit caused by the occlusion of a cerebral blood vessel, resulting in reduced blood flow to brain tissue (Sacco et al., 2013; Musuka et al., 2015; Hui et al., 2022). The pathophysiology of IS has been extensively described in the literature. Ischemia leads to energy failure, calcium overload, and mitochondrial dysfunction, promoting the generation of ROS, protein and lipid oxidation, DNA damage, and activation of apoptotic and necrotic pathways (Chamorro et al., 2016), which contribute directly to neuronal death in the ischemic core and penumbra.

In parallel, ischemic injury triggers neuroinflammation. Damaged cells release DAMPs and interleukins such as IL-1α and IL-33, activating NF-κB signaling and promoting microglial and astrocytic activation. Breakdown of the blood–brain barrier allows infiltration of peripheral immune cells, exacerbating neuronal damage (Bustamante et al., 2016; Qiu et al., 2021; Gao et al., 2023). Systemic factors, including the gut microbiota, modulate these glial responses: dysbiosis can enhance pro-inflammatory T cell activation, which migrates to the brain and amplifies neuroinflammation, while germ-free models show impaired microglial maturation and altered astrocytic signaling, resulting in larger infarcts and worse functional outcomes (Xia and Zhai, 2010; Erny et al., 2015; Singh et al., 2016).

### Evidence of cannabidiol use in stroke models

CBD has shown potential effects in several neurological disorders, including neurodegenerative diseases such as Alzheimer’s disease (Watt et al., 2020; Marques and Campos, 2024) and Parkinson’s disease (Hafida et al., 2024), epilepsy (Borowicz-Reutt et al., 2024), and autism spectrum disorder (Sannar et al., 2024; Costa et al., 2025). Regarding IS, several studies have demonstrated the therapeutic potential of CBD in modulating infarct volume and neurological damage, neuroinflammation, oxidative stress, and excitotoxicity, as well as in regulating the permeability of the BBB and intestinal barrier, in addition to exerting anti-apoptotic effects (Hayakawa et al., 2010; Bigdeli and Khaksar, 2017; Khaksar and Bigdeli, 2017).

It is important to highlight that, following IS, components of the ECS are altered, with increased expression of CB1R and CB2R in the rat brain (Jin et al., 2000; Ashton et al., 2007). CB2R ligands can modulate the inflammatory response with neuroprotective effects, while CB1R activation may induce chemical hypothermia, both being associated with a reduction in infarct volume (Leker et al., 2003; Murikinati et al., 2010). Accordingly, a systematic review and meta-analysis demonstrated that the administration of CB ligands, as well as their modulation by CBD, was able to reduce ischemic lesion volume, with a trend toward infarct reduction even when administered late (up to 6 hours after the ischemic event), in addition to improving neurological scores (England et al., 2015). Another study demonstrated that rats subjected to the MCAO model and pretreated with different doses of CBD for 5 consecutive days showed a significant reduction in infarct volume in the cortex and in the striatum at doses of 100 and 200 ng/rat compared to the vehicle group (Bigdeli and Khaksar, 2017). Since CBD does not directly bind to CB1 and CB2 receptors, some authors suggest that its effects possibly occur through the 5-HT1A receptor and other mechanisms (Mishima et al., 2005; Hayakawa et al., 2010). The reduction in infarct volume, alongside improvements in neurological scores as well as motor and sensory functions, is consistently corroborated by numerous studies (Hayakawa et al., 2004; Ceprián et al., 2017; Rodríguez-Muñoz et al., 2018; Khaksar et al., 2022).

Beyond the modulation of cannabinoid receptors, CBD may exert its neuroprotective effects by attenuating neuroinflammation, a major pathological process following IS (Zarruk et al., 2012; Jayaraj et al., 2019; Vicente-Acosta et al., 2022; Raïch et al., 2024). In particular, the activation of CB2 receptors has been shown to reduce microglial overactivation and the release of pro-inflammatory mediators, while enhancing neuroprotective responses (Zarruk et al., 2012). Additionally, CBD can modulate other molecular targets, including PPAR-γ and TRPV1, contributing to reduced oxidative stress and glial reactivity (Vicente-Acosta et al., 2022; Raïch et al., 2024). It is important to emphasize that the inflammatory response in stroke initially exerts beneficial effects, as it promotes the clearance of cellular debris and aids in the recruitment of cells involved in tissue repair. However, when this response is excessive or prolonged, it becomes detrimental, leading to overactivation of astrocytes and microglia, causing additional neuronal damage, edema, and impaired functional recovery (Jayaraj et al., 2019). In this context, CBD acts as a promising therapeutic agent by reducing excessive glial activation, restoring neuroinflammatory homeostasis, and promoting an environment favorable to neuronal survival and functional recovery (Ceprián et al., 2017; Raïch et al., 2024). Bigdeli and Khaksar (2017) demonstrated that animals subjected to the MCAO model presented the expression levels of tumor necrosis factor receptor 1 (TNFR1) and NF-κB in the whole hemisphere, cortex, and striatum were significantly reduced following administration of CBD at doses of 100 and 200 ng/rat, compared to the vehicle group. TNF binds to TNFR1, triggering intracellular signaling cascades that activate the transcription factor NF-κB, leading to the upregulation of pro-inflammatory genes (Wajant et al., 2003). Notably, a correlation was found between the CBD-induced reduction in infarct volume and decreased expression of TNFR1 and NF-κB in the striatum. Conversely, in the cortex, the reduction in ischemic area was associated with decreased NF-κB expression, but not with TNFR1 (Bigdeli and Khaksar, 2017). Furthermore, CBD has been shown to modulate neuroinflammation by reducing pro-inflammatory molecules in an *in vitro* model of hypoxic-ischemic injury induced by oxygen–glucose deprivation of forebrain slices, partly through mechanisms mediated by CB2 receptors and partly also demonstrating that adenosine is involved in CBD-induced neuroprotection after hypoxia-ischemia, as adenosine receptor antagonists reversed this effect (Castillo et al., 2010). TNF is also known to contribute to apoptotic processes, as it activates caspase-8, ultimately leading to programmed cell death (Wang et al., 2008). In a neonatal stroke model induced by MCAO, animals in the vehicle group exhibited a 50% reduction in the density of surviving neurons within the peri-infarct area. In contrast, CBD treatment preserved neuron-specific nuclear protein (NeuN^+^) cell density and reduced neuronal death, further supporting the neuroprotective properties of CBD, since it also acts by decreasing the B-cell lymphoma 2 and Bcl-2-associated X protein (Bcl-2/Bax ratio) (Ceprián et al., 2017; Khaksar et al., 2022).

**Figure 1:**
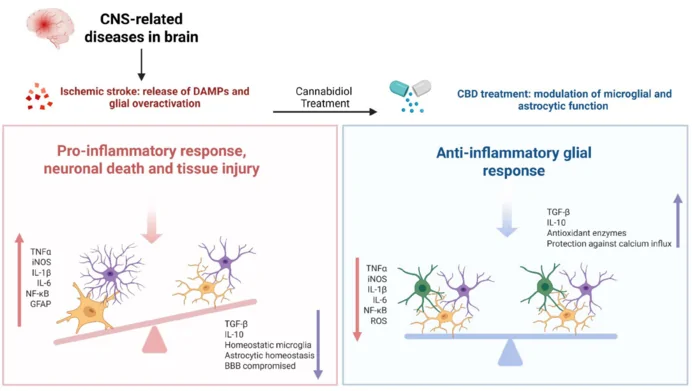
Schematic representation of glial responses in ischemic stroke and the modulatory effect of CBD treatment. On the left, ischemic stroke induces the release of DAMPs and leads to glial overactivation, resulting in a pro-inflammatory response, characterized by increased levels of TNF-α, iNOS, IL-1β, IL-6, NF-κB, and GFAP. These changes contribute to neuronal death, tissue injury, and BBB compromise. On the right, CBD treatment modulates microglial and astrocytic function, promoting an anti-inflammatory glial response. This is evidenced by increased levels of TGF-β, IL-10, antioxidant enzymes, and protection against calcium influx, alongside decreased expression of TNF-α, iNOS, IL-1β, IL-6, NF-κB, and ROS. The balance shifts toward neuroprotection and cellular homeostasis. Created with BioRender.com. BBB: Blood–brain barrier; CBD: cannabidiol; CNS: central nervous system; DAMPs: damage-associated molecular patterns; GFAP: glial fibrillary acidic protein; IL: interleukin; iNOS: inducible nitric oxide synthase; NF-κB: nuclear factor kappa B; ROS: reactive oxygen species; TGF-β: transforming growth factor-beta; TNF-α: tumor necrosis factor-alpha.

Oxidative stress is also one of the key pathophysiological hallmarks of IS. Khaksar et al. (2022) performed a pre-treatment with different doses of CBD in animals subjected to MCAO for 60 minutes. The results indicated a significant increase in the activity of the antioxidant enzyme superoxide dismutase in both the cortex and the striatum, along with enhanced catalase activity. In parallel, elevated levels of malondialdehyde, a marker of lipid peroxidation, were reduced in these same regions. These findings are consistent with another study that also demonstrated the antioxidant role of CBD in young animals (2 months old) subjected to the MCAO stroke model. In that investigation, elevated levels of myeloperoxidase were detected in the heart and lungs, which were attenuated following treatment with a full-spectrum cannabis sativa extract (de Souza Stork et al., 2025). Additionally, the neuroprotective effects of CBD extend to the context of excitotoxicity, in which it appears to modulate calcium-related pathways, including the upregulation of the sodium-calcium exchanger on the plasma membrane (Khaksar and Bigdeli, 2017). During ischemia, excessive glutamate release and activation of N-methyl-D-aspartate receptors promote a massive influx of calcium into the cell, leading to disruption of ionic homeostasis and triggering a cascade of events that culminate in cell death (Obrenovitch and Richards, 1995; Dirnagl et al., 1999). As demonstrated by (Khaksar et al., 2022), animals subjected to IS induced by MCAO showed reduced caspase-3 levels in regions such as the cortex and striatum, along with downregulation of p53, highlighting the anti-apoptotic effects of CBD. These results underscore the ability of CBD to directly modulate oxidative, apoptotic, and excitotoxic processes, which are central contributors to secondary neuronal injury following ischemia. **Figure 1** summarizes CBD effects.

Finally, several studies have demonstrated the potential of CBD in restoring the integrity of the BBB. It is well established that following an ischemic event, the BBB undergoes significant disruption, resulting in increased permeability. However, administration of CBD at doses of 100 and 200 ng/rat significantly attenuated this dysfunction compared to the vehicle group (Khaksar and Bigdeli, 2017). This effect appears to be associated with the inhibition of pro-inflammatory cytokine release, reduced expression of adhesion molecules such as intercellular adhesion molecule 1, and decreased leukocyte migration into the brain parenchyma (El-Remessy et al., 2006; McHugh et al., 2008; Castillo et al., 2010). Complementarily, de Souza Stork et al. (2025) demonstrated that full-spectrum cannabis sativa extract was also able to restore intestinal barrier integrity in a model of is induced by MCAO. This finding supports the hypothesis that post-stroke alterations associated with increased susceptibility to infections may be related to dysfunction in the gut-brain and gut–peripheral organ axes (Hu et al., 2022; Tuz et al., 2022). In this context, other studies have shown that stroke can lead to dysbiosis, promoting increased intestinal permeability and contributing to both inflammatory and infectious outcomes (Chidambaram et al., 2022). All the data are summarized in the **Table 1**.

**Table 1:** Summary of the main neuroprotective effects of cannabidiol in ischemic stroke models, according to the targeted pathophysiological mechanisms

Mechanism/target	Observed effect of cannabidiol	Reference
Infarct volume and neurological function	Reduction in infarct volume; improvement in motor and sensory functions	Bigdeli and Khaksar, 2017; Ceprián et al., 2017; Khaksar et al., 2022
Neuroinflammation	Decreased expression of tumor necrosis factor receptor 1 and nuclear factor-κB; inhibition of pro-inflammatory cytokine release	Castillo et al., 2010; Bigdeli and Khaksar, 2017
Apoptosis	Decrease in Bcl-2/Bax ratio; inhibition of caspase-8 pathway; increased neuronal survival (NeuN^+^)	Ceprián et al., 2017; Khaksar et al., 2022
Oxidative stress	Increased reactive oxygen species and catalase activity; reduction in malondialdehyde levels	Khaksar et al., 2022
Systemic oxidative stress	Reduced myeloperoxidase levels in the heart and lungs	de Souza Stork et al., 2025
Excitotoxicity	Modulation of calcium-related pathways; increased sodium-calcium exchanger expression; protection against excessive calcium influx	Dirnagl et al., 1999; Khaksar and Bigdeli, 2017
Blood–brain barrier integrity	Reduced blood–brain barrier permeability; decreased intercellular adhesion molecule 1 expression; reduced leukocyte migration	McHugh et al., 2008; Castillo et al., 2010; Khaksar and Bigdeli, 2017
Intestinal barrier	Restoration of intestinal barrier integrity	de Souza Stork et al., 2025

### Effects of cannabidiol on glial cell activity in ischemic stroke

Accumulating evidence suggests that CBD exerts protective effects by modulating glial activation and reactivity following cerebral ischemia (Ceprián et al., 2017; Mori et al., 2017; Meyer et al., 2022). Mechanisms illustrated in **Figure 1**. One of the key glial responses to ischemia is the morphological changes of microglia (Matzinger and Kamala, 2011; Muzio et al., 2021; Paolicelli et al., 2022). This alteration is accompanied by increased expression of ionized calcium-binding adapter molecule 1, enlarged soma size, and reduced branch length, features that reflect an inflammatory profile and are associated with the release of pro-inflammatory cytokines (Jin et al., 2010; Kratzer et al., 2014). Several studies have demonstrated that CBD attenuates this microglial activation. In mice subjected to MCAO or photothrombosis models, CBD administration reduced infarct size, decreased total ionized calcium-binding adapter molecule 1 fluorescence and cell counts, and restored a less activated microglial phenotype (Mori et al., 2017; Yokubaitis et al., 2021; Meyer et al., 2022).

Astrocytes also respond robustly to ischemia, particularly in the peri-infarct region, exhibiting increased intracellular Ca^2+^ oscillations in response to neuronal death and danger-associated signals (Meyer et al., 2022; Raïch et al., 2024). These abnormal Ca^2+^ dynamics can exacerbate excitotoxicity and glial reactivity. *In vivo* two-photon imaging revealed that CBD normalized astroglial Ca^2+^ signaling and mitigated peri-infarct astrocyte overactivation following ischemia (Raïch et al., 2024). While the number of glial fibrillary acidic protein (GFAP^+^) astrocytes increased significantly in the peri-infarct cortex in MCAO animals, this effect was attenuated by CBD treatment, though not fully reversed. At 30 days post-ischemia, GFAP expression remained elevated in vehicle-treated animals, whereas CBD-treated rats showed reduced astrogliosis, supporting a role for CBD in modulating long-term gliotic responses (Ceprián et al., 2017).

Mechanistically, these glial effects may involve CB2R modulation, which is known to suppress cytokine release, neutrophil recruitment, and leukocyte adhesion to cerebral vessels (England et al., 2015). CB2R are highly expressed on immune cells, including activated microglia, and their stimulation has been associated with reduced neuroinflammation. Additionally, CBD’s modulation of calcium signaling and indirect actions on 5-HT1A and PPAR-γ receptors may also contribute to its regulation of glial function (Hayakawa et al., 2010). A recent systematic review demonstrated that CBD exhibits remarkable effects in reducing inflammation resulting from microglial and astrocyte activation following IS, acting in a multimodal manner primarily via CB2R, modulating inflammatory cascades and promoting neuroprotective effects by balancing excitatory and inhibitory signals in the brain (Alraddadi et al., 2025). Additionally, a study by Ceprián et al. (2019) demonstrated that animals subjected to the hypoxic-ischemic model and treated with CBD showed preservation of mature oligodendrocytes (Mol) and myelin basic protein (MBP), as well as prevention of functional deficits. These findings reinforce the potential of CBD as a therapeutic agent targeting glial-driven mechanisms in post-stroke neuroinflammation and recovery (England et al., 2015; Yokubaitis et al., 2021; Raïch et al., 2024; Wright, 2024; **Table 2**).

**Table 2:** Effects of cannabidiol on glial cells in preclinical models of ischemic stroke

Mechanism/target	Observed effect of cannabidiol	Reference
Microglia: activation and morphology	Reduction of microglial activation; decreased Iba-1 expression and cell counts; restoration of a less activated phenotype.	Mori et al., 2017; Yokubaitis et al., 2021; Meyer et al., 2022
Microglia: cytokine release	Attenuation of pro-inflammatory cytokine release associated with microglial activation.	Jin et al., 2010; Kratzer et al., 2014; England et al., 2015
Astrocytes: intracellular Ca^2+^	Normalization of abnormal Ca^2+^ oscillations; reduction of peri-infarct astrocyte overactivation.	Meyer et al., 2022; Raïch et al., 2024
Astrocytes: glial fibrillary acidic protein expression	Reduction of astrogliosis; decreased glial fibrillary acidic protein expression in the long term post-ischemia.	Ceprián et al., 2017; Raïch et al., 2024
CB2 Receptors	Modulation of glial activation via CB2R; suppression of cytokine release, neutrophil recruitment, and leukocyte adhesion.	England et al., 2015; Alraddadi et al., 2025
Mature oligodendrocytes, myelin basic protein, functional outcomes	Preservation of mature oligodendrocytes and myelin basic protein, and prevention of neurobehavioral/functional deficits after hypoxia–ischemia.	Ceprián et al., 2019

## Stroke Treatment: Current Approaches and Therapeutic Perspectives of Cannabidiol

Currently, therapeutic approaches for IS focus on restoring cerebral blood flow and limiting tissue damage. The gold-standard treatments are intravenous thrombolysis with tissue plasminogen activator (tPA) and mechanical thrombectomy; however, these interventions must be performed within a 4.5- to 6-hour window after symptom onset (Campbell et al., 2019; Berge et al., 2021; Mosconi and Paciaroni, 2022). In recent years, advances in neuroimaging techniques have allowed the extension of the tPA window to patients with an unknown time of onset or a known onset of up to 9 hours, through the identification of the so-called “tissue window,” which enables the assessment of residual cerebral viability (Berge et al., 2021). Despite these advances, time to treatment initiation remains a key determinant of prognosis, posing a clinical challenge given the frequent delayed hospital arrival of many patients (Darehed et al., 2020; Yafasova et al., 2021).

Although reperfusion is essential to preserve viable brain tissue after IS, it may also exacerbate injury through excessive oxidative mechanisms. The abrupt restoration of blood flow in ischemic areas triggers the overproduction of ROS and reactive nitrogen species (Allen and Bayraktutan, 2009), while the energy depletion during ischemia promotes the accumulation of lactic acid and acidosis (Vexler and Yenari, 2009). This pro-oxidant environment contributes to the formation of highly reactive radicals that damage lipids, proteins, and DNA, thereby amplifying cellular dysfunction, inflammation, and neuronal death (Allen and Bayraktutan, 2009). In this context, the development of therapies aimed at attenuating both the inflammatory response and oxidative stress is crucial to interrupt the pathological cascade that exacerbates ischemic brain damage (Widimsky et al., 2023).

Several molecules have been investigated for the treatment of pathological events associated with IS (Nag et al., 2024). Among them, nicotinamide adenine dinucleotide phosphate oxidase inhibitors, such as apocynin, appear to be capable of reaching the brain tissue and reducing microglial oxidative stress in ischemic lesions (Wang et al., 2006). The immunosuppressant fingolimod, when combined with tPA, regulates the sphingosine 1-phosphate receptor on T lymphocytes, preventing immune cell migration to the inflamed tissue, as well as inhibiting platelet aggregation and reducing the risk of post-stroke hemorrhage. Metformin alone, as well as uric acid in combination with tPA and mechanical thrombectomy, have been shown to improve neurological function and reduce mortality (Chamorro et al., 2017; Zhao et al., 2019).

Preclinical studies with intravenously administered minocycline (1 mg/kg) in rat models of IS demonstrated a reduction in TNF levels and an increase in heat shock protein 70 and human antigen R expression in the ischemic penumbra. Furthermore, the reduction of inflammation in the injured area translated into a significant improvement in motor performance (Pawletko et al., 2023). Complementary clinical studies indicate that oral minocycline treatment in individuals who suffered IS improves neurological deficits at 30 and 90 days compared with controls (Lampl et al., 2007). Additionally, PPAR-γ agonists, such as rosiglitazone and artemisinin, have been shown to reduce brain tissue loss and improve sensorimotor and cognitive functions up to 21 days after ischemia, as well as alleviate short- and long-term neurological deficits and exert anti-inflammatory effects on microglial cells, promoting neurogenesis (Han et al., 2015; Li et al., 2025).

Although various drugs and molecules are being investigated to mitigate brain alterations following IS, CBD stands out for its ability to act on multiple targets, as detailed in **Tables 1** and **2**. Evidence indicates that this phytocannabinoid exerts neuroprotective effects by modulating glial activation and reactivity after cerebral ischemia. It reduces microglial activation, promoting a less inflammatory phenotype, decreasing soma size, and maintaining cellular ramification, which is associated with reduced release of pro-inflammatory cytokines (Mori et al., 2017; Yokubaitis et al., 2021; Meyer et al., 2022). Furthermore, CBD modulates astrocyte reactivity, normalizing Ca^2+^ signaling patterns and attenuating peri-infarct astrocyte hyperactivity, thereby helping to limit excitotoxicity and long-term gliotic processes (Ceprián et al., 2017; Raïch et al., 2024). These glial effects appear to involve CB2 receptor modulation, which suppresses cytokine release and leukocyte adhesion, as well as indirect actions on 5-HT1A and PPAR-γ receptors, reinforcing therapeutic potential of CBD in regulating post-stroke neuroinflammation and promoting functional recovery (Hayakawa et al., 2010; England et al., 2015; Wright, 2024).

## Gaps and Challenges in Clinical Translation

Despite advances in the understanding of stroke pathophysiology, effective treatment remains a major challenge. Recent studies have focused on identifying effective strategies for the development of neuroprotective agents. However, no neuroprotective drug has yet been clinically proven or made available (Qiang et al., 2022). Among the difficulties in translating the use of CBD are the variations in the timing of its administration, whether before or after the injury (Belardo et al., 2019; Santiago-Castañeda et al., 2022) as well as the challenge of selecting the most appropriate route of administration, since there is still no established standard (Millar et al., 2018).

Additionally, the wide variety of *Cannabis*-based formulations used in studies, including pure CBD isolates, whole-plant extracts, and preparations enriched with other cannabinoids such as low-dose THC, represents a significant obstacle to result comparability and experimental reproducibility (Russo, 2011). This issue is further compounded by the lack of standardization regarding a safe and effective therapeutic dose, as well as the limited understanding of potential drug interactions associated with CBD use (Gaston and Friedman, 2017; Millar et al., 2019).

Furthermore, most of the available evidence comes from preclinical studies in animal models, which, although they provide valuable insights into mechanisms of action and biological effects, cannot be directly extrapolated to humans, serving only as a guiding foundation for future clinical investigations (de Oliveira et al., 2024). Differences in metabolism, pharmacokinetics, and bioavailability between rodents and humans can influence the effective dose and therapeutic window. The complexity of human stroke, including comorbidities, age, and variability in lesion size and location, is not fully captured in experimental models (Ujváry and Hanuš, 2016; Sommer, 2017; Popa-Wagner et al., 2018). Most preclinical studies also use controlled timing and routes of CBD administration that may not translate directly to clinical practice (England et al., 2015; Sommer, 2017). These factors emphasize the need for dose optimization, pharmacokinetic evaluation, and well-designed clinical trials to assess the efficacy and safety of CBD in human stroke patients. Considering these limitations, there is a clear need for rigorous clinical trials with standardized and translational designs that can validate the efficacy and safety of CBD. Overcoming these challenges is essential for this phytocannabinoid to move from a preclinical therapeutic promise to an effective intervention in the clinical management of IS.

Finally, although the literature on the effects of CBD on glial cells in the context of IS is limited, it is important to highlight that most studies focus on the potential therapeutic effects of CBD. The scarcity of negative or null studies does not reflect a bias in study selection, but rather a gap in the current research. The review by Carter et al. (2024) discusses that cannabis use may increase the risk of IS, especially in young users, and that the negative effects may depend on the frequency and duration of use. However, it is crucial to differentiate the effects of CBD, which is an isolated compound from cannabis, from those of the whole plant.

## Conclusion

CBD has emerged as a promising therapeutic agent due to its ability to modulate glial cell activity, reduce neuroinflammation, and protect against oxidative stress in IS. By targeting astrocytes, microglia, and oligodendrocytes, CBD promotes neuroprotection and functional recovery in preclinical models. Despite encouraging findings, clinical translation remains limited, underscoring the need for standardized studies and well-designed trials to confirm its efficacy and safety in human stroke patients.

## Data Availability

*Not applicable.*
